# *Gad67* haploinsufficiency reduces amyloid pathology and rescues olfactory memory deficits in a mouse model of Alzheimer’s disease

**DOI:** 10.1186/s13024-017-0213-9

**Published:** 2017-10-10

**Authors:** Yue Wang, Zheng Wu, Yu-Ting Bai, Gang-Yi Wu, Gong Chen

**Affiliations:** 1 Department of Biology, Huck Institutes of Life Sciences, Pennsylvania State University, University park, PA 16802 USA; 2 School of Life Science, South China Normal University, Guangzhou, 510631 China

**Keywords:** Alzheimer’s disease, GAD67, Haploinsufficiency, Amyloid beta, Astrocytic GABA, Tonic inhibition, Neuroinflammation, Microglia, Olfactory memory

## Abstract

**Background:**

Alzheimer’s disease (AD) is the most common age-related neurodegenerative disorder, affecting millions of people worldwide. Although dysfunction of multiple neurotransmitter systems including cholinergic, glutamatergic and GABAergic systems has been associated with AD progression the underlying mechanisms remain elusive. We and others have recently found that GABA content is elevated in AD brains and linked to cognitive deficits in AD mouse models. The glutamic acid decarboxylase 67 (GAD67) is the major enzyme converting glutamate into GABA and has been implied in a number of neurological disorders such as epilepsy and schizophrenia. However, whether Gad67 is involved in AD pathology has not been well studied. Here, we investigate the functional role of GAD67 in an AD mouse model with *Gad67* haploinsufficiency that is caused by replacing one allele of *Gad67* with green fluorescent protein (*GFP*) gene during generation of GAD67-GFP mice.

**Methods:**

To genetically reduce GAD67 in AD mouse brains, we crossed the *Gad67* haploinsufficient mice (GAD67-GFP^+/−^) with 5xFAD mice (harboring 5 human familial AD mutations in APP and PS1 genes) to generate a new line of bigenic mice. Immunostaining, ELISA, electrophysiology and behavior test were applied to compare the difference between groups.

**Results:**

We found that reduction of GAD67 resulted in a significant decrease of amyloid β production in 5xFAD mice. Concurrently, the abnormal astrocytic GABA and tonic GABA currents, as well as the microglial reactivity were significantly reduced in the 5xFAD mice with *Gad67* haploinsufficiency. Importantly, the olfactory memory deficit of 5xFAD mice was rescued by *Gad67* haploinsufficiency.

**Conclusions:**

Our results demonstrate that GAD67 plays an important role in AD pathology, suggesting that GAD67 may be a potential drug target for modulating the progress of AD.

**Electronic supplementary material:**

The online version of this article (doi: 10.1186/s13024-017-0213-9) contains supplementary material, which is available to authorized users.

## Background

Alzheimer’s disease (AD) severely impacts the life quality of millions of people around the world. As the most common aging-related dementia, AD is characterized by accumulation of extracellular amyloid β peptide (Aβ) deposits and declining in cognition and memory [1]. Dysregulation of several neurotransmitter systems have been reported in the disease progress. GABAergic system is the principal inhibitory neurotransmitter system in the brain. Diverse lines of evidence suggest that GABAergic system is altered in AD brains, including GABA [2, 3], GABA synthetase, GABA receptors [4], GABA transporters [3] and GABAergic neurons [5, 6]. For example, the activity of GABAergic neurons in AD mice is significantly reduced [5], and even GABAergic neurons may be lost in AD brains [7, 8]. On the other hand, we and others have discovered that GABA may accumulate in the reactive astrocytes in both human and mouse AD brains [2, 4]. Therefore, GABA system may have a distinct role in AD pathology.

GABA is the major inhibitory neurotransmitter in the adult brain, and is converted from glutamate by glutamic acid decarboxylase (GAD). In mammalian brains, GAD contains two isoforms, GAD65 and GAD67 [9]. GAD65 is mainly localized at the presynaptic nerve terminals, while GAD67 is distributed throughout the cell. Importantly, over 90% of the basal GABA in the brain is synthesized by GAD67 [10, 11]. Accordingly, *Gad67* knock-out mice will die within a week after birth, but *Gad67* haploinsufficient mice are viable although with abnormal behaviors [12]. In contrast, *Gad65* knockout mice are largely normal, but exhibiting susceptibility to seizures [13]. Interestingly, dysfunction of GAD67 has been associated with several neurological disorders, including schizophrenia [14], bipolar disorders [15] and Parkinson’s disease [16]. In fact, clinical trials targeting at GAD67 for the treatment of Parkinson’s disease have achieved some success [17]. For AD, it is reported that GAD67 is not changed in brain tissues of postmortem AD patients, but whether *Gad67* is involved in the progress of AD is largely unknown.

We investigated the functional role of GAD67 in AD by reducing *Gad67* through crossing GAD67-GFP knock-in mice with 5xFAD mice, which carry 5 human familial AD mutations in APP and PS1 genes [18]. Here, we found that the Aβ plaques were significantly reduced by genetic downregulation of GAD67 in 5xFAD brains. Moreover, excessive microglia-mediated neuroinflammation, abnormal astrocytic GABA and excessive GABA tonic inhibition were also largely reduced in 5xFAD brains with *Gad67* haploinsufficiency. Importantly, the olfactory memory deficit in the 5xFAD mice was rescued in the bigenic mice with *Gad67* haploinsufficiency. Therefore, *Gad67* haploinsufficiency in AD mouse model shows significant improvement in both AD pathology and cognitive function. Our results suggest that GAD67 may potentially be a drug target for AD therapy.

## Methods

### Experimental animals

The GAD67-GFP knock-in mice in a C57BL/6 genetic background were obtained from (Tamamaki et al., 2003). A cDNA encoding *Gfp* was targeted to the *Gad67* promoter locus and the resulting homozygous mice die prenatally but the heterozygous GAD67^+/−^-GFP mice, in which one allele of *Gad67* gene is replaced by *Gfp*, are viable and seem have no global morphological and overt behavioral deficits [19, 20] but see some social behavior changes [12]. The 5xFAD transgenic mice also in a C57BL/6 genetic background, were purchased from the Jackson Lab (stock number: 006554), which harbored two mutations on human PS1 proteins (M146 L and L286 V) and three mutations on human APP [Swedish (K670 N/M671 L)], London (V717I) and Florida (I716V). This transgenic line was crossed with the C57BL/6 for breeding. To assess the effects of reduction of GAD67 in AD mouse model, we generated the compound mouse lines by crossing female GAD67+/−GFP mice with male 5xFAD mice, thus producing the following four genotypes: wild type (AD-GG-), GAD67-GFP^+/−^ (AD-GG+), 5xFAD (AD+GG-) and 5xFAD/GAD67-GFP^+/−^ (AD+GG+, bigenic mice) respectively. Of note, the 5xFAD mice in C57BL/6 J genetic background do not show very early onset. (2–3 month old) amyloid pathology and behavioral deficits as the original line generated by the Vassar group in a B6SJL background [Jackson Lab, https://www.jax.org/strain/006554]; therefore, we assessed the effects of haploinsufficiency of *Gad67* on amyloid pathology and behavioral deficits in 6–10 months old mice, when the 5xFAD mice developed severe Aβ burden and consistent behavioral deficits. The 5xFAD transgene is driven by a Thy-1 promoter, which contains an estrogen responsive element, and is known to display a gender difference in Aβ burden [21]; thus, we kept the female/male ratio comparable cross the 4 genotypes. For immunostaining age- and sex-matched littermates were used. For behavior test, several cohorts with similar age were pooled together for comparison.

The mice were housed in a 12/12-h light-dark cycle with food and water. All experimental procedures were approved by the Pennsylvania State University IACUC, in line with the guidelines of the National Institutes of Health for the Care and Use of Laboratory Animals.

### Immunohistochemistry and quantification

Mice were deeply anesthetized by intraperitoneally injected with 2.5% Avertin (10 ml/kg), and then were perfused with ice-cold artificial cerebrospinal fluid (ACSF) to wash off blood in the brain, followed by 4% paraformaldehyde (PFA) fixation in phosphate-buffered saline (PBS). The brain was dissected out and further fixed overnight at 4 °C with 4% PFA. Brain tissue was sliced as sagittal sections at 45 um using the Leica vibratome and stored in 0.1 M PB at 4 °C. For immunostaining, brain slices were rinsed with PBS for three times, 10 min each, and then treated with blocking solution (0.3% Triton-X and 10% normal donkey and goat serum in 0.1 M PBS) for 2 h at room temperature. The brain sections were next incubated with the following primary antibodies at 4 °C overnight [in 5% normal donkey serum (NDS) and 5% normal goat serum (NGS) in 0.1 M PBS]: Monoclonal anti-GAD67 (mouse, 1:500, Millipore, MAB5406); polyclonal anti-GABA (rabbit, 1:1000, Sigma, A2052); Monoclonal anti-Parvalbumin (mouse, 1:2000, Sigma, P3088); Rabbit monoclonal anti-beta Amyloid 1–42 (rabbit, 1:2000, Invitrogen, 700,254); Polyclonal anti-Glial Fibrillary Acidic Protein (chicken, 1:1000, Millipore, AB5541); polyclonal anti-Iba1 (rabbit, 1:500, Wako, 019–19,741); monoclonal anti-iNOS (mouse, 1:500, BD, 610,328). After washing three times with PBS, the brain sections were then incubated with appropriate secondary antibodies conjugated to Alexa Fluor 488 (1:1000, Jackson ImmunoResearch), Cy3(1:500, Jackson ImmunoResearch) or Alexa Fluor 647 (1:1000, Jackson ImmunoResearch) for 2 h at room temperature. After three times of washing using PBS, the brain sections were mounted on slides with the antifading mounting solution with DAPI (Invitrogen by Thermo Fisher Scientific, P36931).

For thioflavin-s staining, the brain slides were firstly treated following the immunohistochemistry protocol described above. After tissue samples were incubated in secondary antibody, the samples were firstly washed by diluted thioflavin-s in PBS (2 μg/ml) for 10 min on the shaker. Then wash the tissue sample with PBS for twice, 10 min each time. Samples were mounted using ProLong Gold antifade reagent (Life Technologies, P36934) on slides.

Fluorescent images were acquired with a Keyence microscope (BIOREVO BZ9000 viewer & analyzer) or an Olympus confocal microscope (FV 1000). The confocal acquisition parameters were set at the same level for each of the individual protein immunostaining (see Additional file 1 Table S1). The mean intensity of each of the immunostaining marker was analyzed by the ImageJ software at the same setting (ImageJ 1.46r, Wayne Rasband, National Institutes of Health, USA).

### Human Aβ Elisa

Frontal cortex was isolated from AD+GG- and AD+GG+ littermate mice, and were further lyzed with NP40 cell lysis buffer (Invitrogen, FNN0021) containing protease inhibitor cocktail (Sigma-Aldrich, P2714) and 1 mM PMSF protease inhibitor (Thermo Scientific, 36,978). The lyzed tissue samples were centrifuged at 13,000 rpm at 4 °C. The supernatant was collected for ELISA experiment. Aβ42 and Aβ40 measurement were conducted by using the human Aβ42 ELISA kit (Invitrogen, KHB3441) and human Aβ40 ELISA kit (Invitrogen, KHB3481), and following the protocol of the ELISA kits strictly. Firstly, to bind antigen, load standards and samples to the corresponding wells in the Aβ antibody coated plates (included in the ELISA kit). Secondly, add the human Aβ42 detector antibody (or human Aβ40 detector antibody, respectively), tap the plate to mix thoroughly and then incubate for 3 h at room temperature on a shaker. Next, aspirate the solution and wash the wells with 1× wash buffer for 4 times. Add the HRP-conjugate antibody, incubate the plate at room temperature for 30 min, and then washed the wells 4 times with 1× wash buffer. Then apply the stabilized chromogen in each well, incubate in the dark for 30 min. After adding the stop solution in each well, read the plate within 30 min to get the absorbance at 450 nm and then generate the standard curve (SpectraMax Plus 384 Microplate Reader). With the value of optical density at 450 nm and the known concentration of the standard ladder, the Aβ42 and Aβ40 load of the unknown samples will be analyzed and quantified. Each sample is repeated twice and averaged.

### Brain slice electrophysiology

The adult brain slice preparation followed previously described protocols [22]. Briefly, the 12–14 month old adult mice were transcardially perfused with cutting solution (in mM): 93 NMDG, 93 HCl, 2.5 KCl, 1.25 NaH_2_PO_4_, 30 NaHCO_3_, 20 HEPES, 15 Glucose, 12 N-Acetyl-L-cysteine, 5 Sodium ascorbate, 2 Thiourea, 3 Sodium pyruvate, 7 MgSO_4_, 0.5 CaCl2, pH 7.3–7.4, 300 mOsmo, bubbled with 95% O_2_/5% CO_2_. Then, the mouse brain was removed and cut at 300 μm in the cutting solution at room temperature. Brain slices were collected in the cutting solution and incubated for 12–15 min at 32–34 °C. The slices were kept in the holding solution with continuous 95% O_2_/5% CO_2_ bubbling (in mM): 92 NaCl, 2.5 KCl, 1.25 NaH_2_PO_4_, 30 NaHCO_3_, 20 HEPES, 15 Glucose, 12 N-Acetyl-L-cysteine, 5 Sodium ascorbate, 2 Thiourea, 3 Sodium pyruvate, 2 MgSO_4_, 2 CaCl_2_. After 0.5 h recovery in the holding solution, patch-clamp recording was performed in the standard aCSF (in mM): 124 NaCl, 2.5 KCl, 1.25 NaH_2_PO_4_, 26 NaHCO_3_, 10 Glucose, 1.3 MgSO_4_, 2.5 CaCl_2_.

To record the miniature IPSCs and the tonic GABA currents, 10 μM DNQX, 50 μM AP5 and 1 μM TTX were added into the aCSF. The pipette was filled with high Cl^−^ internal solution (mM): 135 CsCl, 5 Na-phosphocreatine, 10 HEPES, 5 EGTA, 4 MgATP, and 0.5 Na2GTP (pH 7.3 adjusted with CsOH, 280–290 mOsm). The tonic GABA current was revealed by the change of holding current after local perfusion of 100 μM bicuculline (sigma). The mIPSCs were analyzed by Mini Analysis Program and assisted with visual check. Data were collected with a MultiClamp 700A amplifier and pCLAMP9 software (Molecular Devices).

### Olfactory behavior test

Olfactory deficits of mice were tested using the odor habituation and cross dis-habituation test as described previously [23]. Briefly, four neutral odors [Ethyl valerate (#290866), 2-Heptanone (#537683), Isopentyl acetate (#112674), (R)-(+)-Limonene (#183164); Sigma Aldrich, St. Louis, MO] were diluted 1 × 10^-3^ in mineral oil respectively. For each odor, a cotton applicator stick was firstly dipped in the odor solution and then enclosed inside a plastic tube to prevent a direct contact of liquid odor to the testing chamber. The four odors were randomly presented by inserting the odor stick into a port on the side of the mouse home cage, in 4 successive trials, 20 s each, and separated by 30 s inter-trial intervals. The mouse was caging individually in this odor test to prevent interference from social interaction with other mice. The olfactory behavior test was performed during the light phase of light-dark cycle (12 h/12 h). An observer who was blinded to the genotypes of the animals during test and data analysis recorded the investigation time, which was defined as snout-oriented sniffing time within 1 cm of the odor presentation port [23]. For the measurement of investigation time, the duration time each mouse sniffs the odors presented in different trials were recorded. For investigation time (normalized) were measured by the investigation time (sec) in each trial of odor investigation divided by the sniffing time spent in the mouse’s first trial in the same odor presentation. The investigation time and normalized investigation time were then analyzed using two-way ANOVAs followed by Bonferroni post-tests. The cross-habituation index was calculated by the average of subtracting the normalized fourth trial odor investigation value from the following normalized first trial odor investigation value. The cross-habituation index was further analyzed by one-way ANOVAs followed by LSD post hoc test, compare all pairs of columns.

### Data analysis

Data were represented as mean ± s.e.m. Student’s t-test was used for statistical analysis in two-group comparison. One-way ANOVA or two-way ANOVA analysis was used for comparison among multiple groups. Statistical significance was set at *p* < 0.05. In the olfactory behavior test, for the calculation of F and *P* value of cross-habituation index, analysis the data using two - way ANOVA with LSD post hoc test via SPSS software. Statistical significance was set at *p* < 0.05, labeled as *. *p* < 0.01, labeled as **. *p* < 0.001, labeled as ***. All behavioral tests and analyses were performed blindly.

## Results

### GAD67 is reduced in GAD67-GFP knock-in mice

**Fig. 1:**
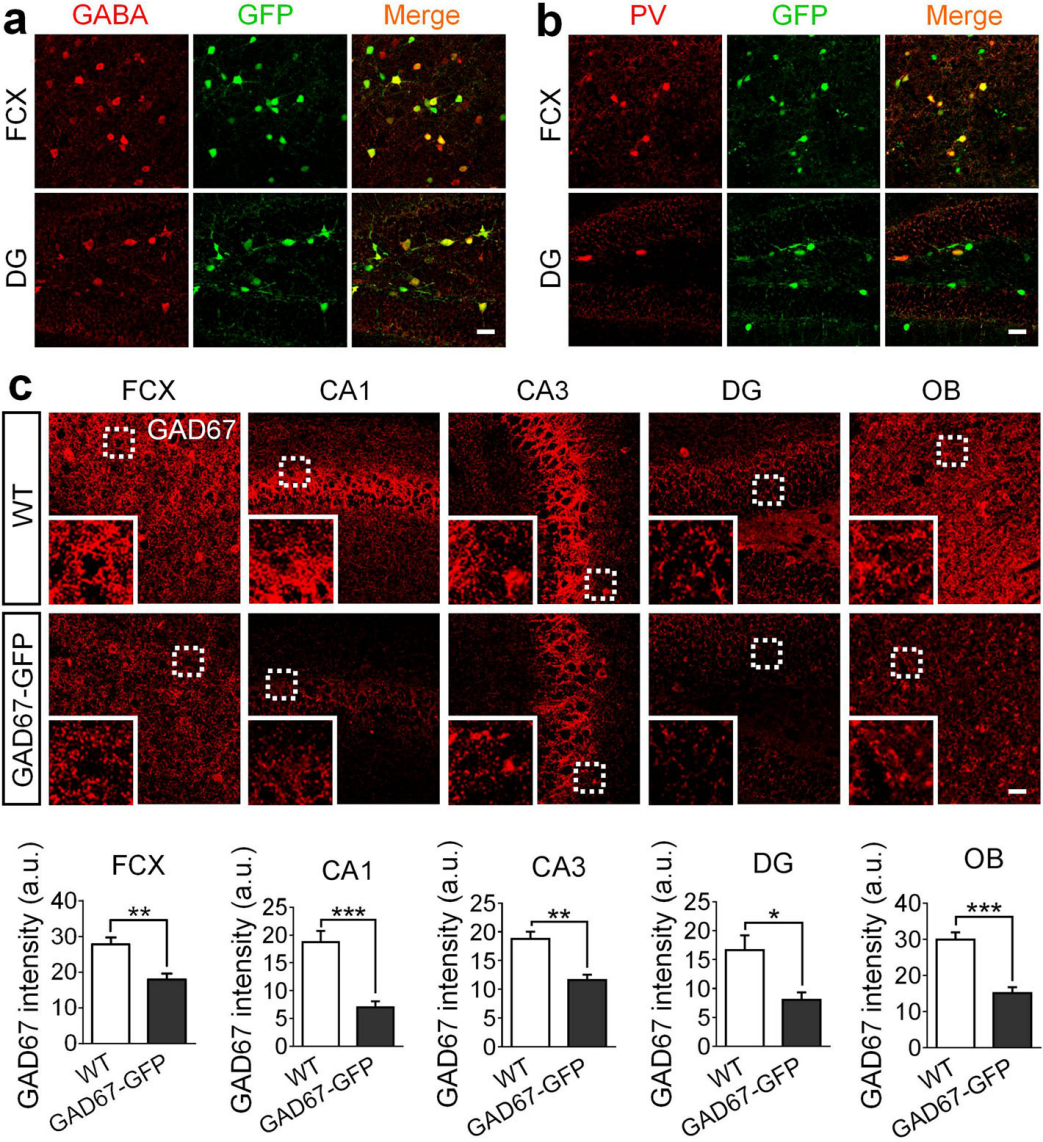
Characterization of the GAD67-GFP knock-in transgenic mice. **a** Most GABA immunopositive cells (red) are also GFP positive (green) in both frontal cortex (FCX) and dentate gyrus (DG) in hippocampus of the adult GAD67-GFP knock-in mice. **b** In frontal cortex and dentate gyrus of hippocampus, all of the parvalbumin (PV) positive cells (one subtype of GABAergic neurons) (red) are labeled by GFP (green). **c** Representative confocal micrographs display the expression level of GAD67 (red) in GAD67-GFP knock-in mice and GFP-negative mice of 10–11 months old. Dotted line circled regions were zoomed in and were shown in the lower left corner of each micrograph. Quantification data showing GAD67 intensity in frontal cortex (FCX), CA1, CA3, dentate gyrus (DG) and olfactory bulb (OB) of GAD67-GFP knock-in mice is significantly reduced to about half of the GAD67 content level in WT controls. For a-c, scale bars represent 30 μm. *N* = 5 WT mice and 5 GAD67-GFP knock-in mice. Data are presented as mean ± s.e.m., * *p *< 0.05; ** *p *< 0.01; *** *p *< 0.001; Student’s t-test

GAD67 is a major enzyme for producing GABA in the brain. Previous study has generated a GAD67-GFP transgenic mouse line [20], where *GFP* gene was knocked into the *Gad67* gene locus so that GABAergic neurons will express GFP under the control of *Gad67* promoter. However, the knock-in of GFP also destroyed the expression of GAD67. Therefore, the heterozygous GAD67-GFP mice had only one copy of *Gad67*, resulting in haploinsufficiency of *Gad67*. The homozygous GAD67-GFP mice were lethal, suggesting that GAD67 is critical for normal brain function. We first confirmed that the GAD67-GFP transgenic mice indeed had reduced GAD67 level. As expected, most of the GFP-positive cells (>95%) were GABA-immunopositive neurons (Fig. 1a). Moreover, a subset of GFP-positive cells were also immunopositive for parvalbumin (PV), a typical marker for a subtype of GABAergic neurons (Fig. 1b). These data demonstrate that GFP is a reliable reporter for GABAergic neurons in the GAD67-GFP mice.

Because one allele of *Gad67* is replaced by *Gfp* in the heterozygotes (haploinsufficiency), we next compared the expression level of GAD67 between GAD67-GFP^+/−^ and wildtype (WT) littermates. Consistent with previous finding that GAD67 was significantly reduced in the heterozygous GAD67-GFP^+/−^ mice [20], we also found that the immunoreactivity of GAD67 was reduced approximately by half in various brain regions in the heterozygotes (Fig. 1c). Thus, GAD67-GFP knock-in mice provide a unique transgenic mouse model for investigating the functional role of GAD67 in neurological disorders.

### *Gad67* haploinsufficiency alleviates the amyloid β burden in 5xFAD mouse brains

**Fig. 2:**
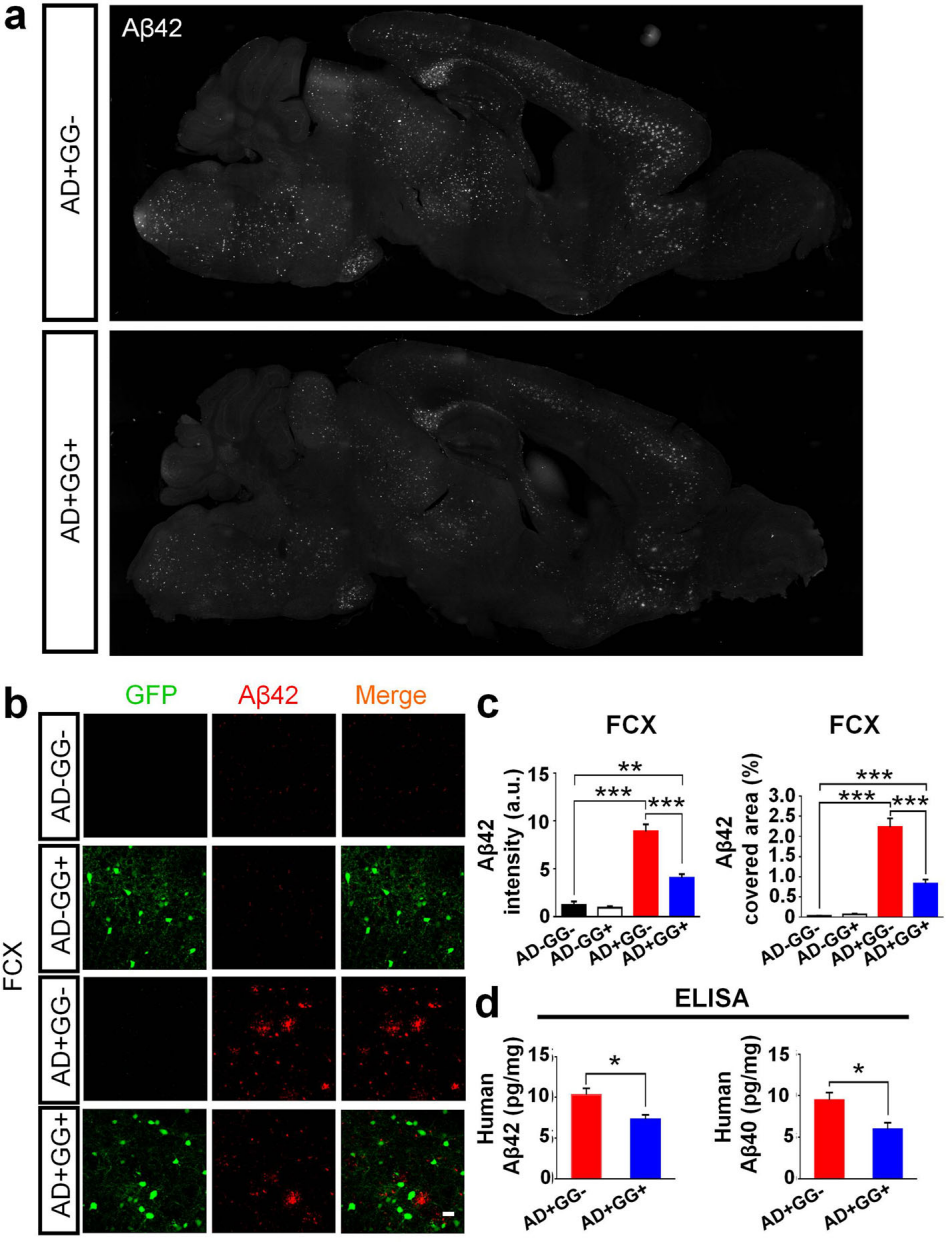
A significant reduction of amyloid-beta plaques in the *Gad67* haploinsufficiency 5xFAD mouse brains. **a** Images of the sagittal section of whole brain showing decreased Aβ42 deposits (white) in AD+GG- (top) and AD+GG+ (bottom) mice of 10–11 months age. **b** Representative confocal images display the level of Aβ42 deposits (red) in the four genotypes (AD-GG-, AD-GG+, AD+GG-, AD+GG+) generated by crossing of GAD67-GFP knock-in mice with 5xFAD mice. GFP cells showed in green. Scale bar = 30 μm. **c** Quantification analysis showing a remarkable reduction of Aβ42 (left panel and right panel, Aβ42 intensity and Aβ42 covered area percentage, respectively) in the bigenic (AD+GG+) mice brain than 5xFAD mice of similar age. *N* = 6 mice of 10–11 months age in each genotype groups (AD-GG-, AD-GG+, AD+GG-, AD+GG+). **d** Elisa data also showing the Aβ40 and Aβ42 were significantly reduced in 5xFAD mice by Gad67 haploinsufficiency (unpaired Student’s *t* test). Data are presented as mean ± s.e.m., * *p *< 0.05; ** *p *< 0.01; *** *p *< 0.001; one-way ANOVA with the Tukey’s post-hoc test when comparing multiple groups

To investigate the effect of genetic reduction of GAD67 on Alzheimer's disease (AD), we crossed the 5xFAD transgenic mice with GAD67-GFP^+/−^ heterozygotes to generate four different genotypes of littermates: wild type (AD-GG-), GAD67-GFP^+/−^ (AD-GG+), 5xFAD (AD+GG-) and 5xFAD/GAD67-GFP^+/−^ (AD+GG+, bigenic mice). We first tested the effect of reducing GAD67 on the amyloid β (Aβ) plaques, the hallmark for AD [24]. Consistent with previous findings [18, 25], we found that 5xFAD mice (AD+GG-) developed severe Aβ42 deposits across many brain regions (Fig. 2a, top). However, the Aβ42 level appeared to be reduced significantly in the bigenic mice (AD+GG+) with *Gad67* haploinsufficiency (Fig. 2a, bottom) (age 10–11 months). For example, Aβ42 immunostaining signal accumulated at a high level in the frontal cortex (FCX) of AD+ mice, and quantitative analysis revealed that both Aβ42 intensity and covered area were significantly decreased in AD+GG+ mice compared to the AD+GG- mice (Fig. 2b and c). Besides immunostaining, we further employed an Aβ ELISA kit (Aβ40 and Aβ42) to examine the Aβ production level in 5xFAD mouse brains with *Gad67* haploinsufficiency. Consistent with our Aβ42 immunostaining results, the ELISA test also showed a significant reduction of Aβ42 (Fig. 2d; AD+GG-: 10.32 ± 0.79, *n* = 6; AD+GG+: 7.31 ± 0.55, n = 6, *p *< 0.02, Student’s *t* test), as well as Aβ40 (Fig. 2d; AD+GG-: 9.47 ± 0.91, n = 6; AD+GG+: 5.95 ± 0.80, n = 6, *p *< 0.02; Student’s *t* test) in the AD+GG+ FCX homogenates. Therefore, Aβ production in the 5xFAD mouse brains is downregulated by *Gad67* haploinsufficiency.

**Fig. 3:**
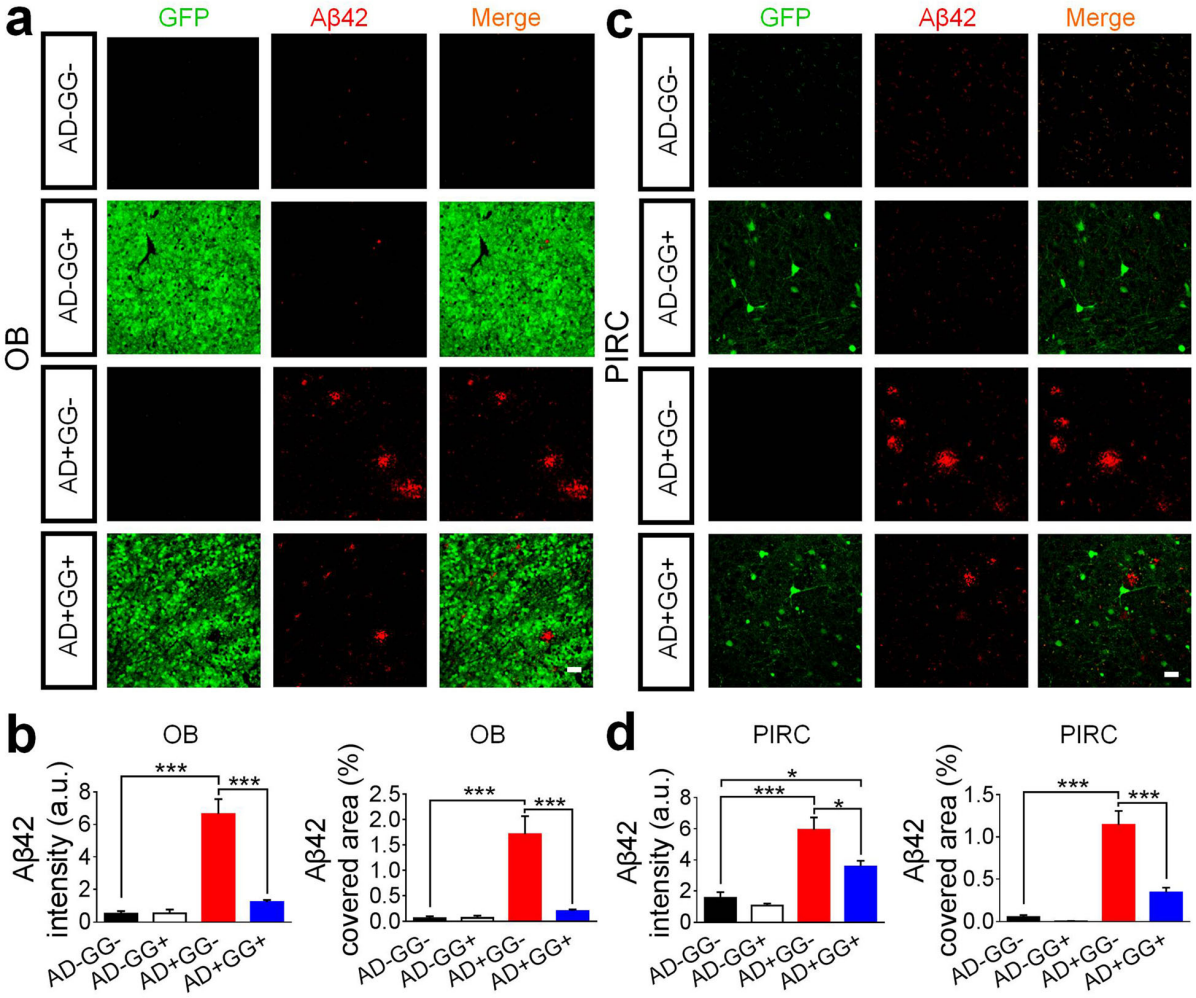
Changes of amyloid-beta burden in brain regions related olfactory function**. (a-d)** *Gad67* haploinsufficiency 5xFAD mice (AD+GG+) also elicit a reduction of Aβ42 burden (red) in olfactory bulb (OB) (**a-b**) and piriform cortex (PIRC) (**c-d**). GFP positive cells were shown in green. N = 6 mice of 10–11 months age in each genotype groups (AD-GG-, AD-GG+, AD+GG-, AD+GG+). For **a** and **c**, scale bars represent 30 μm. Data are presented as mean ± s.e.m., * *p *< 0.05; ** *p *< 0.01; *** *p *< 0.001; one-way ANOVA with the Tukey’s post-hoc test when comparing multiple groups

We further analyzed the Aβ42 level in the olfactory bulb (OB) and the piriform cortex (PIRC) in mice with different genetic background (Fig. 3). Consistently, the Aβ42 deposit was significantly increased in both OB (Fig. 3a) and PIRC (Fig. 3c) in the AD+ mice compared to AD- mice (age 10–11 months). Interestingly, among AD+ mice, the GG+ mice showed a significant reduction of Aβ42 in the OB and PIRC compared to the GG- mice (Fig. 3a and c, bottom two rows). Quantitative analysis confirmed a significant decrease of Aβ42 in both OB and PIRC of bigenic mice (AD+GG+) compared to the 5xFAD littermates (AD+GG-) (Fig. 3b and d). We have also quantified the Aβ42 level in the hippocampus, which showed slight reduction in AD+GG+ mice (Aβ42: AG+GG-, 2.89 ± 0.80 vs AD+GG+, 1.18 ± 0.13, *n* = 3, *p *> 0.1; Student’s *t* test), but it’s not as significant as in the cortical areas. We noted a significant low level of Aβ42 in the hippocampus compared to that in the frontal cortex in 5xFAD brains. One potential reason for such difference may be due to different GABAergic neuron population in the hippocampus versus the cortical regions, including the total number of GABAergic neurons and the composition of various subtypes.

In addition to Aβ42 immunostaining, we further employed thioflavin-s staining to verify our findings in the bigenic mice. Thioflavin-s is a chemical dye widely used to visualize Aβ aggregates and hyperphosphorylated tau tangles [26]. We observed a significant reduction of thioflavin-s labeled plaques in the FCX and PIRC of the bigenic mice (AD+GG+) compared to the AD+GG- mice (Additional file 1: Figures S1). Accompanying the changes of Aβ plaques, we also observed a significant reduction of neuronal density surrounding the Aβ plaques in the FCX of 5xFAD mice (10–11 months old), but such neuronal loss was partially rescued in bigenic mice (AD+GG+) (Additional file 1: Figure S2), suggesting that *Gad67* haploinsufficiency not only reduces amyloid plaques but also preserves surrounding neurons in the 5xFAD mouse model. Taken together, these data demonstrate an unexpected role of *Gad67* haploinsufficiency in alleviating the Aβ deposit and its detrimental effect on neurons in the 5xFAD mouse brains.

### *Gad67* haploinsufficiency reduces astrocytic GABA in 5xFAD mouse brains

**Fig. 4:**
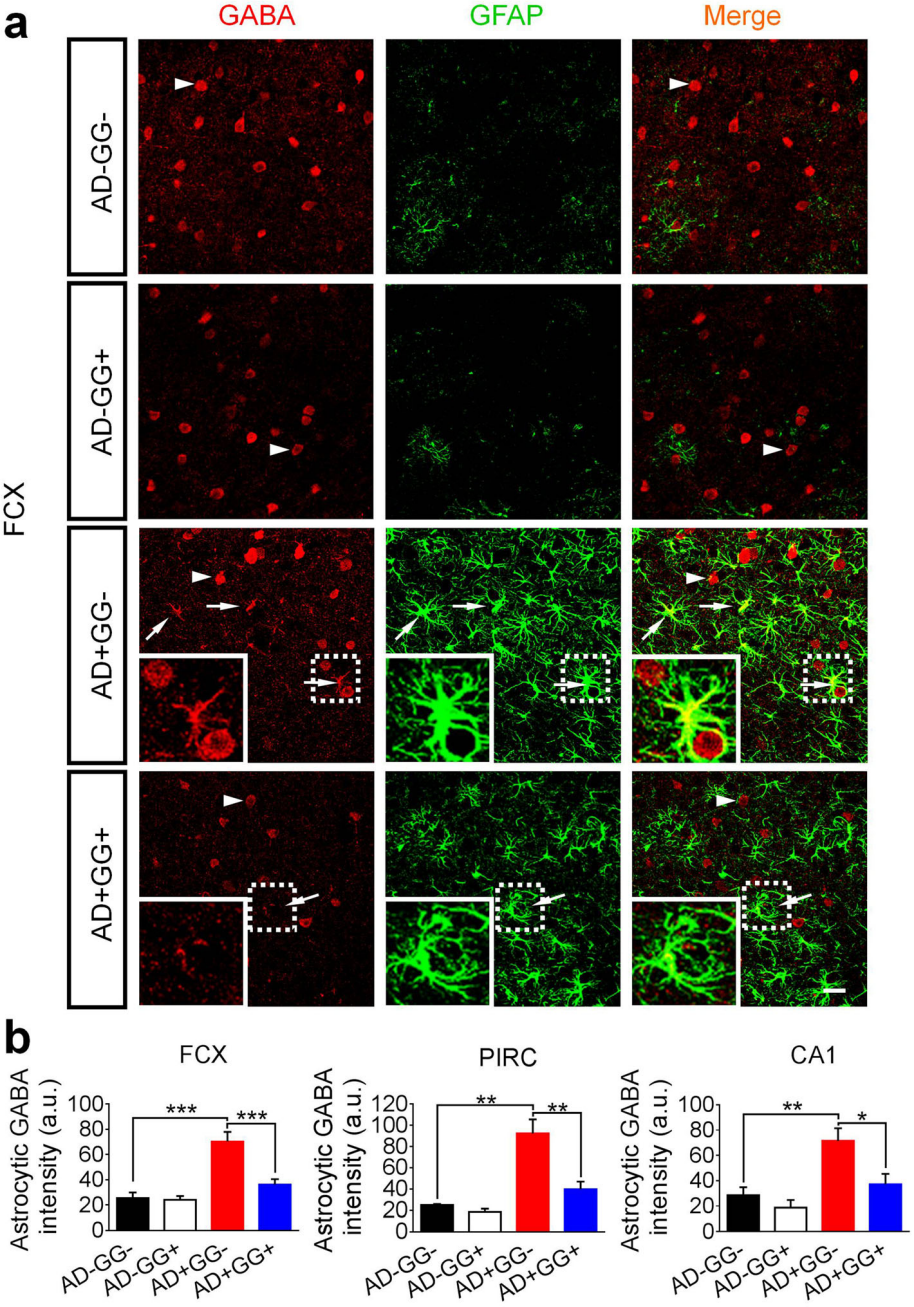
Mitigation of the abnormal astrocytic GABA in 5xFAD mice with *Gad67* haploinsufficiency. **a** The GABA content level (red) is abnormally enhanced in astrocytes (pseudo colored in green for merge purpose) in 5xFAD mice brain while it is under detectable level in AD-negative mice (the normal condition). Notably, the astrocytic GABA content level is significantly reduced in *Gad67* haploinsufficiency 5xFAD brain. Arrowhead: GABA positive cells (red). Arrow: astrocytic GABA (red). Dotted line circled regions were zoomed in and were shown in the lower left corner of each micrograph. Scale bar represents 30 μm. **b** Quantification data showing the significant reduction of astrocytic GABA in frontal cortex (FCX), piriform cortex (PIRC) and CA1 region of the bigenic (AD+GG+) brain than the 5xFAD mice of similar age. N = 6 mice of 10–11 months age in each genotype groups (AD-GG-, AD-GG+, AD+GG-, AD+GG+). Data are presented as mean ± s.e.m., * *p * < 0.05; ** *p * < 0.01; *** *p * < 0.001; one-way ANOVA with the Tukey’s post-hoc test when comparing multiple groups

Recently, we and others have found that GABA content is elevated in AD mouse brains and in particular, an abnormal astrocytic GABA has been detected in both AD mouse model and postmortem human AD brain tissue [2, 3]. Since GAD67 is a major enzyme for producing GABA in the brain, we examined whether the astrocytic GABA level in AD mice was altered by *Gad67* haploinsufficiency. Brain sections of all four genotype littermate mice (AD-GG-, AD-GG+, AD+GG-, and AD+GG+; sex- and age-matched for each genotype) at 10–11 months old were double immunostained with GABA and astrocyte marker GFAP. Consistent with previous findings, we found a high level of GABA in the reactive astrocytes of the 5xFAD mice (AD+GG-), but not non-AD mice (AD-GG- and AD-GG+) (Fig. 4a). Interestingly, the abnormal astrocytic GABA was significantly reduced in the bigenic mice with *Gad67* haploinsufficiency (AD+GG+) compared to normal 5xFAD mice (AD+GG-) (Fig. 4a, bottom two rows). Quantitative analyses in the FCX, PIRC, and hippocampal CA1 regions all confirmed a uniform reduction of astrocytic GABA in AD+GG+ mice (Fig. 4b). In contrast to the astrocytic GABA change, we observed no significant change of neuronal GABA level across all four groups of mice (Additional file 1: Figures S3), suggesting that neuronal GABA was not affected by *Gad67* haploinsufficiency in the adult mice, consistent with previous study [20]. Together, our data suggest that the abnormal astrocytic GABA in AD mouse brains can be largely alleviated through genetic reduction of GAD67.

### *Gad67* haploinsufficiency decreases abnormal tonic GABA currents in 5xFAD mice

**Fig. 5:**
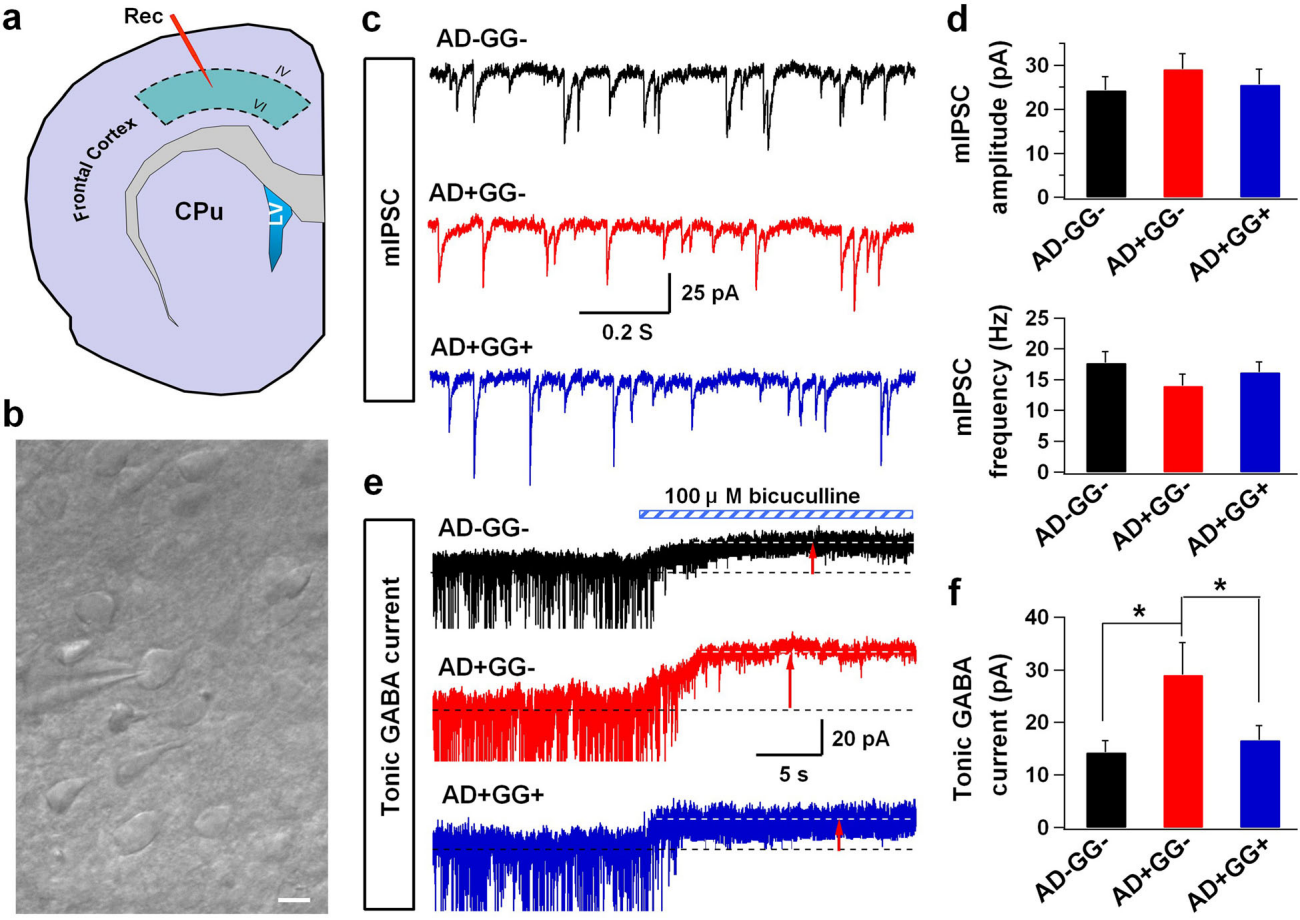
Abnormal tonic inhibition is decreased in 5xFAD mice with *Gad67* haploinsufficiency. **a** Schematic image illustrate the interesting cortical area for electrophysiology study. **b** A typical phase contrast image showing the whole cell recording in the adult brain slice. Scale bar = 10 μm.﻿ **c** Example of the mIPSCs traces in AD-GG-, AD+GG- and AD+GG+ groups. **d** Summary graph of mean amplitude and frequency of mIPSCs. **e** Representative trances of tonic GABA currents. **f** Quantification data showing the abnormal tonic GABA currents in 5xFAD mice are decreased by *Gad67* haploinsufficiency. Data are presented as mean ± s.e.m., * *p* < 0.05; one-way ANOVA with the LSD post-hoc test

Previous studies reported that tonic inhibition is increased in AD mouse brains due to excessive GABA accumulation in the reactive astrocytes [2, 3], or in mouse brains after focal stroke [27]. Since we observed a significant reduction of astrocytic GABA in the 5xFAD brains after genetic reduction of GAD67, we wondered whether tonic GABA currents might also be reduced correspondingly. Whole-cell patch-clamp recordings were performed on the layer IV-VI cortical neurons (Fig. 5a and b). Consistent with our previous findings, tonic GABA currents were significantly increased in the 5xFAD mice (AD+GG-) compared to AD-GG- control littermates (Fig. 5e and f; AD-GG-, 14.4 ± 2.3 pA, *n* = 9 from 2 mice; AD+GG-, 29.1 ± 6.1 pA, *n* = 8 from 2 mice; *p*< 0.02; One-way ANOVA). Interestingly, the abnormal tonic GABA currents in 5xFAD mouse brains were significantly reduced by *Gad67* haploinsufficiency (Fig. 5e and f; AD+GG+, 16.6 ± 2.8 pA, *n* = 15, from 2 mice, *p *< 0.03). On the other hand, there were no obvious differences in the miniature inhibitory postsynaptic currents (mIPSCs) among different groups (Fig. 5c and d). Thus, our results demonstrate that *Gad67* haploinsufficiency results in a reduction of the abnormal GABA tonic currents in cortical neurons of 5xFAD mice.

### Microglia reactivity is ameliorated by *Gad67* haploinsufficiency

**Fig. 6:**
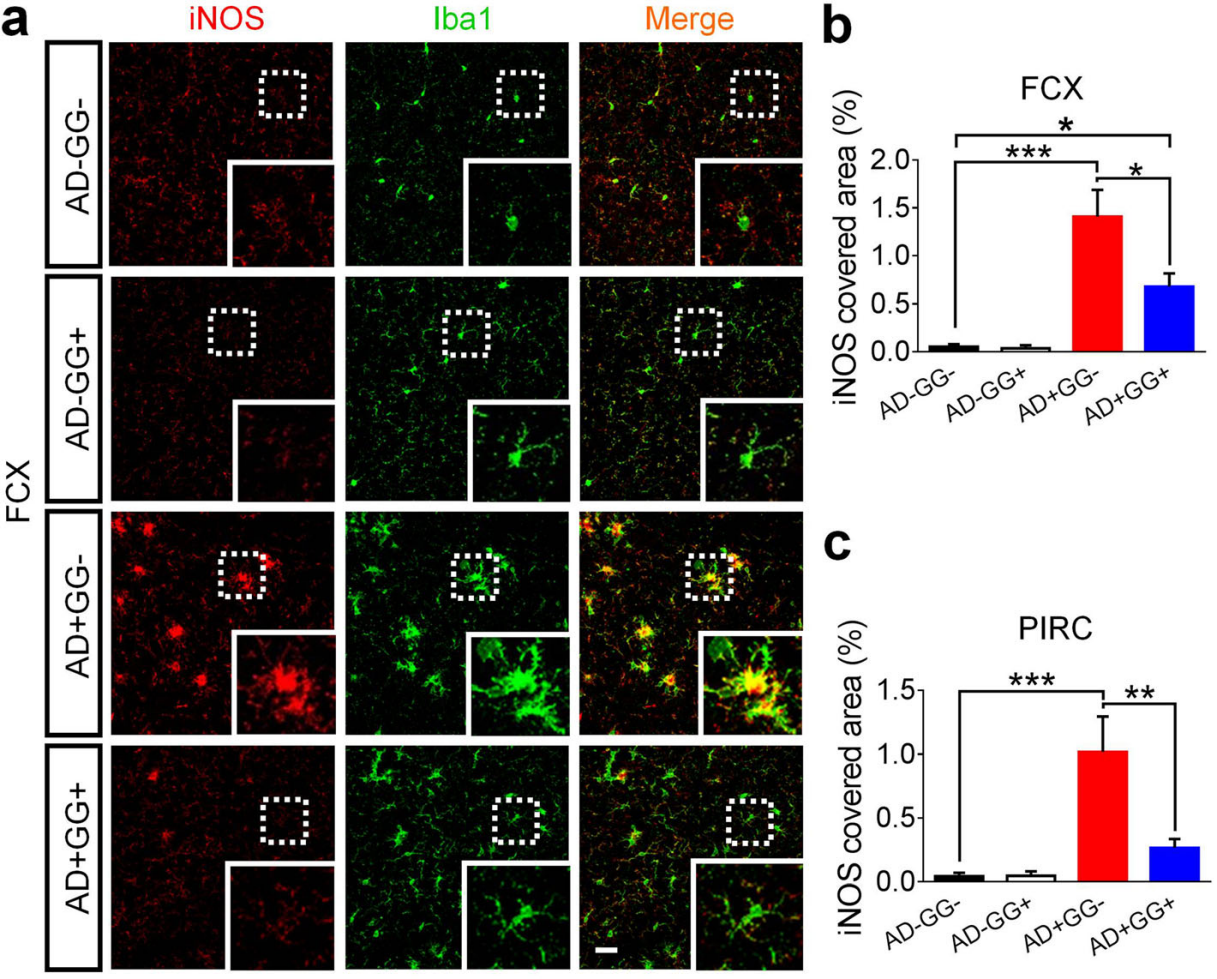
Neuroinflammation mediated by microglia is alleviated in 5xFAD mice with *Gad67* haploinsufficiency. **a** The iNOS positive immune cells (red), as the pro-inflammatory subtype of microglia (pseudo colored in green for merge purpose), is remarkably reduced in frontal cortex (FCX) and piriform cortex (PIRC) of bigenic brain (AD+GG+), close to the level in AD negative brain (normal condition). Dotted line circled regions were zoomed in and were shown in the lower right corner of each micrograph. Scale bar represents 30 μm. **b-c** Quantification analysis displays a dramatic reduction of iNOS covered area percentage in both frontal cortex and piriform cortex of AD+GG+ than AD+GG- of similar age. N = 6 mice of 10–11 months age in each genotype groups (AD-GG-, AD-GG+, AD+GG-, AD+GG+). Data are presented as mean ± s.e.m., * *p* < 0.05; ** *p* < 0.01; *** *p* < 0.001; one-way ANOVA with the Tukey’s post-hoc test when comparing multiple groups

Previous study has suggested that microglia activity is regulated by GABA released from astrocytes [28]. Since the astrocytic GABA was dramatically decreased in 5xFAD mice with *Gad67* haploinsufficiency (Fig. 4), we investigated whether microglia activity in 5xFAD mice might also be changed correspondingly. For this purpose, we investigated the inducible nitric oxide synthase (iNOS), a widely used marker for proinflammatory reactive microglia, in 5xFAD mice (Fig. 6a). In non-AD mice (AD-GG- and AD-GG+), iNOS was not detectable in microglia labeled by Iba1; but in AD+GG- mice, strong iNOS immunoreactivity was detected (Fig. 6a). Surprisingly, in AD+GG+ mice, the iNOS immunoreactivity was significantly reduced compared to their AD+GG- littermates (Fig. 6a). Quantitative analysis shows that in both the frontal cortex (Fig. 6b) and piriform cortex (Fig. 6c), the iNOS covered area was significantly reduced in AD+GG+ mice compared to that in AD+GG- mice (Fig. 6b, FCX, AD+GG-: 1.40 ± 0.28%, AD+GG+: 0.68 ± 0.14%, *p *< 0.02, *n* = 6; Fig. 6c, PIRC, AD+GG-: 1.02 ± 0.28%, AD+GG+: 0.27 ± 0.07%, *p *< 0.01, n = 6). Consistent with the iNOS finding, the resting microglia (Iba1+) displayed normal morphology with small soma and ramified processes, and evenly distributed in various brain regions in AD- mice (Fig. 6a). In contrast, in AD+GG- mouse brains, microglia was strongly activated, exhibiting hypertrophic soma with amoeboid-like morphology (Fig. 6a). Notably, in AD+GG+ mice, the microglia became less hypertrophic with more ramified processes compared to those found in AD+GG- mice, suggesting that the microglia reactivity in 5xFAD mice was partially rescued by GAD67 haploinsufficiency (Fig. 6a, Iba1+, green). Together, these results suggest that genetic reduction of GAD67 partially ameliorates microglia reactivity in 5xFAD brains.

### *Gad67* haploinsufficiency rescues olfactory deficits in 5xFAD mice

**Fig. 7:**
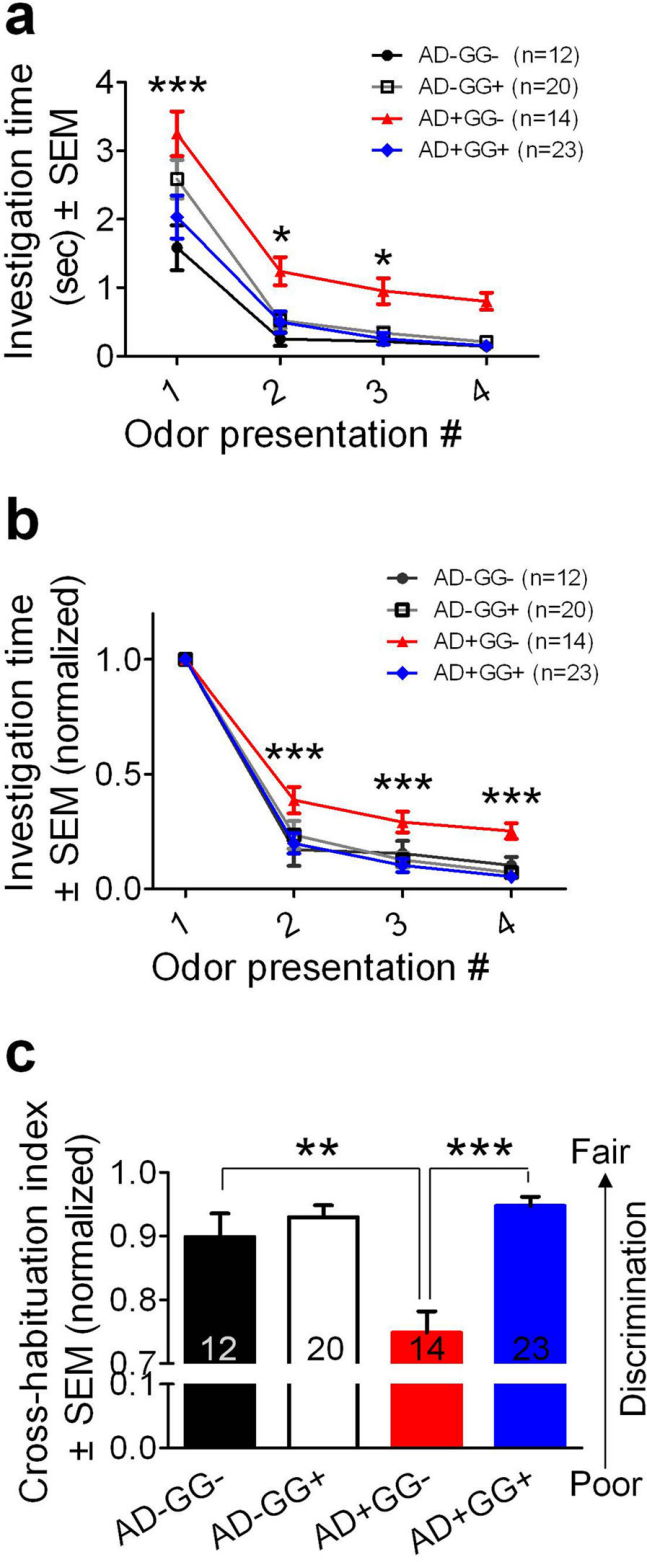
Rescue of olfactory deficit in 5xFAD mice with *Gad67* haploinsufficiency. Olfactory behavior test is applied to examine the extent of olfactory sensory impairment in the four genotypes of 6–8 months age (AD-GG-, AD-GG+, AD+GG-, AD+GG+). **a** Statistical graph showing the bigenic (AD+GG+) mice displayed a reduction of investigation time in each odor presentation trials than 5xFAD mice (red line). **b** Quantification data showing the AD+GG+ mice displayed a reduction of investigation time (normalized) (blue line) than 5xFAD mice (red line). Notably, AD+GG+ mice displayed almost recovery of the impairment olfactory sensory function (which is a common pathological symptom in 5XFAD), according to the result that the statistic values are very close to that of AD-negative mice (black line: AD-GG-; grey line: AD-GG+). **c** AD+GG+ displayed a recovered cross-habituation index (mean ± s.e.m., normalized). For (A)-(C), *n* = 12 AD-GG-, 20 AD-GG+, 14 AD+GG-, 23 AD+GG+ mice of 6–8 months age. * *p* < 0.05; ** *p* < 0.01; *** *p* < 0.001; two-way ANOVA followed by LSD post hoc test for comparison among multiple groups for multiple conditions

We found that several beneficial effects of *Gad67* haploinsufficiency in 5xFAD mice were observed in various brain regions, including the key regions of olfactory circuits and limbic systems as described above. Olfaction sensory impairment has been reported both in AD patients and in mouse models [29, 30]. Therefore, we further investigated whether *Gad67* haploinsufficiency might rescue olfactory deficits in 5xFAD mice. We performed the odor habituation and cross dis-habituation behavior tests as described in previous studies [23, 31]. Briefly, mice were exposed to four novel neutral odors presented in their home cages, and each odor was delivered across four successive trials. The investigation time for each of the 4 odors across four successive trials was recorded and quantified for olfactory habituation. Typically, when first exposed to an odor stimulus, mice would spend longest investigation time but quickly lost interest when exposed 2nd, 3rd, and 4th time to the same odor (Fig. 7a and b, black line). The 5xFAD mice (AD+GG-) displayed consistent impairments in olfactory habituation, showing prolonged investigation time even after repeated exposure to the same stimulus (Fig. 7a and b, red line). Interestingly, the olfactory deficit of AD+GG- was rescued in 5xFAD mice with *Gad67* haploinsufficiency (AD+GG+) (Fig. 7a and 7b, blue line), consistent with the reduction of Aβ in the olfactory bulb and piriform cortex of AD+GG+ mice. Similarly, AD+GG- mice exhibited a significant impairment in cross-habituation test, which was also rescued by *Gad67* haploinsufficiency (Fig. 7c, AD-GG-: 0.90 ± 0.04; AD+GG+: 0.95 ± 0.02, *p *> 0.05, *n* = 12 AD-GG-, 20 AD-GG+, 14 AD+GG-, 23 AD+GG+). Because we did not observe sex differences in olfactory behavioral tests (Additional file 1: Figures S4), data from both sexes were pooled and quantified together (Fig. 7)**.** Two-way ANOVA analysis revealed that both genotypes and successive trials had a significant effect on the investigation time of olfactory habituation (genotype: F (3260) = 12.0; *p* < 0.001; successive trials: F (3260) = 9.0; *p* < 0.001). There was no interaction between genotypes and successive trials (F (9260) = 0.27, *p* > 0.05). Together, our results indicate that *Gad67* haploinsufficiency rescues olfactory deficits in 5xFAD mice.

## Discussion

In this study, we unexpectedly found that when 5xFAD mice were crossed with GAD-GFP mice that destroyed one allele of *Gad67*, the Aβ load was significantly decreased throughout many brain regions. Further investigation revealed that accompanying such Aβ reduction, the astrocytic GABA and tonic inhibition were significantly reduced, and microglial reactivity was also alleviated. More importantly, the olfactory memory deficit of AD mice was partially rescued by *Gad67* haploinsufficiency. These results suggest that GAD67 plays an important role in modulating Aβ production and may provide an important drug target for AD treatment.

### Aβ reduction in 5xFAD mice with *Gad67* haploinsufficiency

As the principal inhibitory neurotransmitter in the brain, GABA plays a critical role in maintaining excitation/inhibition balance, which is important for information processing in the brain. Imbalance of excitation/inhibition has been implicated in a wide range of neurological and neuropsychiatric disorders including AD [32]. GABA is synthesized from excitatory neurotransmitter glutamate by two distinct but partial redundant glutamic acid decarboxylase isoforms, GAD65 and GAD67. Mice with genetic deletion of *Gad65*, which is mainly localized in the synaptic terminals, are viable and develop normally without obvious changes in the basal GABA transmission and brain GABA content, except an increase in seizure susceptibility [13, 33]. In contrast, GAD67 is largely cytosolic and distributed evenly throughout the cells. Deletion of *Gad67* leads to perinatal death and more than 90% reduction of GABA content [20, 34]. In addition to neuronal GABA function, several recent studies including our own have started to investigate non-neuronal changes of GABA system (review see [35, 36]). In particular, we and others have shown that reactive astrocytes activated by Aβ in 5xFAD mice can accumulate a high amount of GABA, which can lead to exuberant tonic GABA inhibition and contribute to memory deficits [37]. Here, we demonstrate that genetic down-regulation of GAD67 dramatically reduces the reactive astrocytic GABA content, as well as Aβ burden and inflammatory microglia in 5xFAD mice, further suggesting a strong link between astrocytic GABA and AD pathology.

There are now compelling evidences supporting that abnormal astrocytic GABA is associated with disease status such as brain injury and neurodegenerative disorders (for review, see [36]). However, the source(s) and mechanism(s) of astrocytic GABA release are still in debate. Previous reports have shown that human astrocytes have all of the machineries for GABA synthesis, metabolism and release, including GAD67, gamma-aminobutyric acid transaminase (GABAT), glia specific GABA transporter GAT3/4, and functional GABA_A_ and GABA_B_ receptors [28, 38]. We found that astrocytic GABA in Alzheimer’s brain was synthesized by astrocytic GAD67 and released by astrocyte-specific GABA transporter GAT3/4 [3]. At the same time, another study reported that glial GABA is synthesized through an unconventional pathway by monoamine oxidase B and released via bestrophin 1 (Best1) channel to mediate tonic inhibition [2]. Unfortunately, the clinic trial of MAO-B inhibitor for Alzheimer’s treatment has failed in phase IIb clinical trial. Here, we provide in vivo genetic evidence showing that *Gad67* haploinsufficiency can significantly alleviate the abnormal astrocytic GABA in a mouse model of AD, underscoring a major role of GAD67—the conventional GABA biosynthesis pathway—in AD pathology.

While the precise mechanisms of GAD67 in AD require further study, one possible hypothesis based on our results is that genetic reduction of GAD67 reduces astrocytic GABA level in AD brains, corrects abnormal neuronal activity, and reduces Aβ production. Consistent with our hypothesis, knockdown of GAD67 protein level has been reported to normalize neuronal activity in a rat model of Parkinson’s disease [39]. In AD mouse models, the generation of Aβ is found coupled with neuronal activity [40, 41], and Aβ pathology can promote neuronal hyperexcitation [5]. Therefore, neuronal activity and Aβ pathology may form a positive feedback loop to boost each other. Disrupt such loop may provide an alternative strategy to delay AD progression. For example, gamma oscillation is a normal rhythmic brain activity important for learning and memory, but it is disrupted in AD mouse brains. Recovery of this rhythmic gamma oscillation not only reduces the Aβ production but also rescues the memory deficits in an AD mouse model [5, 42]. Since >90% GABA is synthesized by GAD67, and GABA is the major inhibitory neurotransmitter, it is possible that *Gad67* haploinsufficiency will regulate GABA production both in neurons and astrocytes, which in turn will modulate neuronal activity during AD progression.

### Abnormal tonic GABA currents and AD

Tonic GABA currents are mediated by extrasynaptic GABA_A_ receptors with a high affinity for GABA and result in a persistent GABAergic inhibition, which is involved in several brain diseases including epilepsy, stroke and Alzheimer’s disease [2, 3, 27, 43]. In AD brains, tonic inhibition is upregulated because of the activation of extrasynaptic GABA_A_ receptors by astrocytic GABA release [2, 3], but synaptic GABA_A_ receptor-mediated phasic inhibition is not changed significantly [2, 5]. Here, we found that the abnormal GABA accumulation in the reactive astrocytes of 5xFAD mouse brains was remarkably reduced by *Gad67* haploinsufficiency, and the abnormal tonic inhibition was also decreased correspondingly, consistent with the reduction of Aβ production and amelioration of microglial reactivity in the 5xFAD mice with *Gad67* haploinsufficiency. Our finding of GAD67 in regulating AD pathology is also supported by earlier studies demonstrating a strong link between gamma oscillation activity (20–80 Hz) and AD [5, 42] as well as tonic GABA inhibition [44, 45].

### Microglia reactivity and AD

The characteristic ramified morphology of microglia and astrocytes and their close relationship with neurons put glial cells in a unique position to sense and maintain a homeostatic microenvironment in the brain (for review see [46]). In recent years, growing evidence has started to unravel an active role of microglia in neuroinflammation, a defense mechanism mediated primarily by reactive microglia and astrocytes [47–49]. However, direct interventions with neuroinflammation have failed in the clinical trials for AD, underscoring the importance of further investigation of the molecular mechanisms of AD pathogenesis. Here, we made an important discovery that aberrant astrocytic GABA may be closely linked to microglia-mediated proinflammatory signaling iNOS and amyloidogenesis. Our findings open a new avenue for therapeutic intervention for AD. Interestingly, consistent with our finding of GAD67 in AD pathology, GABA has been reported to play a pro-neuroinflammatory role in a mouse model for multiple sclerosis [50]. Therefore, this newly discovered link among astrocytic GABA, microglial reactivity, and Aβ production may offer new insight into the complex AD pathology. It is likely that a cocktail approach addressing multiple targets such as neurons, glial cells, and Aβ together, rather than a single drug acting on a single target, will be future solutions to treat complicated syndromes such as AD.

## Conclusion

In contrast to the traditional view that GABAergic transmission is relatively well-preserved in AD patients, growing evidences have demonstrated more complex changes of the GABAergic system in AD pathology in recent years [35, 51]. However, direct interventions with either GABA agonists or antagonists have failed in several human studies and clinical trials, raising the pressing needs for a further investigation of the underlying mechanisms and better intervention strategies. Here, by genetically targeting GAD67, an activity-regulated major GABA synthesis enzyme, we show that *Gad67* haploinsufficiency in AD mouse brains has significant beneficial effects on both pathological and behavioral outcomes. Although the precise mechanisms of how aberrant astrocytic GABA is linked to pro-inflammatory microglia and amyloidogenesis still requires further investigation, our study provides a new perspective for understanding the relationship between GABAergic functions and AD pathogenesis. Together with our previous finding of astrocytic GABA in AD brains, our results support the notion that GAD67 may be a potential target for developing a new treatment for AD.

## Additional file

Additional file 1: Table S1. Confocal imaging acquisition parameters. **Figure S1.** Reduction of Aβ aggregates labeled by thioflavin-s in the frontal cortex (FCX) and piriform cortex (PIRC) of 5xFAD mice with GAD67 haploinsufficiency. **Figure S2.** Immunohistochemistry revealed less neuron loss in the frontal cortex region of 5xFAD mice with *Gad67* haploinsufficiency. **Figure S3.** Neuronal GABA content level remains similar in the frontal cortex among different genotypes of mice. **Figure S4** Similar performance between male and female mice of the same genotype in the olfactory behavioral test. (DOCX 1610 kb)

## Abbreviations

AD: Alzheimer’s disease
Aβ: β-Amyloid
FAD: Familiar Alzheimer’s disease
FCX: Frontal cortex
GABA: Gamma-Aminobutyric acid
GAD: Glutamic acid decarboxylase
GFP: Green fluorescent protein
GG: GAD67-GFP^+/−^
iNOS: Inducible nitric oxide synthase
OB: Olfactory bulb
PIRC: Piriform cortex
