# Supplementary material for: Gad67 haploinsufficiency reduces amyloid pathology and rescues olfactory memory deficits in a mouse model of Alzheimer’s disease
Source: Mol Neurodegener. 2017 Oct 10;12:73. doi: 10.1186/s13024-017-0213-9 (PMC5633901; doi:10.1186/s13024-017-0213-9)
Supplement: Additional file 1: Table S1. — Confocal imaging acquisition parameters. Figure S1. Reduction of Aβ aggregates labeled by thioflavin-s in the frontal cortex (FCX) and piriform cortex (PIRC) of 5xFAD mice with GAD67 haploinsufficiency. Figure S2. Immunohistochemistry revealed less neuron loss in the frontal cortex region of 5xFAD mice with Gad67 haploinsufficiency. Figure S3. Neuronal GABA content level remains similar in the frontal cortex among different genotypes of mice. Figure S4 Similar performance between male and female mice of the same genotype in the olfactory behavioral test. (DOCX 1610 kb) [file 13024_2017_213_MOESM1_ESM.docx]

***Gad67* haploinsufficiency reduces amyloid pathology and rescues olfactory behavior deficits in a mouse model of Alzheimer’s disease**

Yue Wang^1^, Zheng Wu^1,^*, Yu-Ting Bai^1^, Gang-Yi Wu^1,^*, Gong Chen^1,^*

**Supplementary Figures and Legends:**


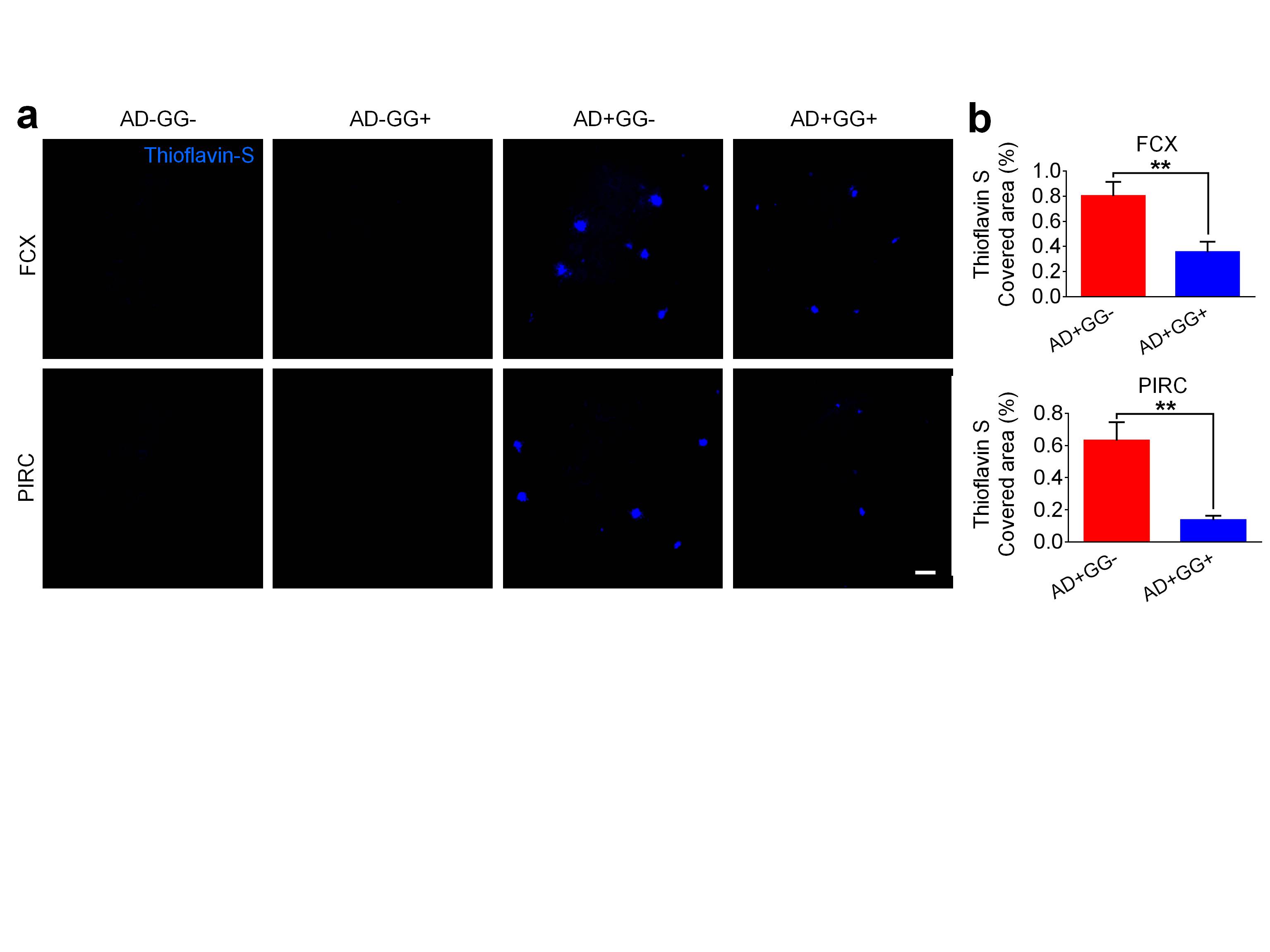


**Figure S1 Reduction of Aβ aggregates labeled by thioflavin-s in the frontal cortex (FCX) and piriform cortex (PIRC) of 5xFAD mice with GAD67 haploinsufficiency.** **a** Representative confocal images displayed the thioflavin-s positive aggregates (blue) in FCX and PIRC of AD+GG- and AD+GG+ mice brain, which is absent in AD negative mice brains. Scale bar represents 30 μm. **b** Quantification data showing the significantly reduction of thioflavin-s covered area percentage in FCX and PIRC in AD+GG+. N = 6 mice of 10-11 months age in each genotype group (AD-GG-, AD-GG+, AD+GG-, AD+GG+). Data are presented as mean ± s.e.m., * *p* < 0.05; ** *p* < 0.01; *** *p* < 0.001; Student’s t-test.


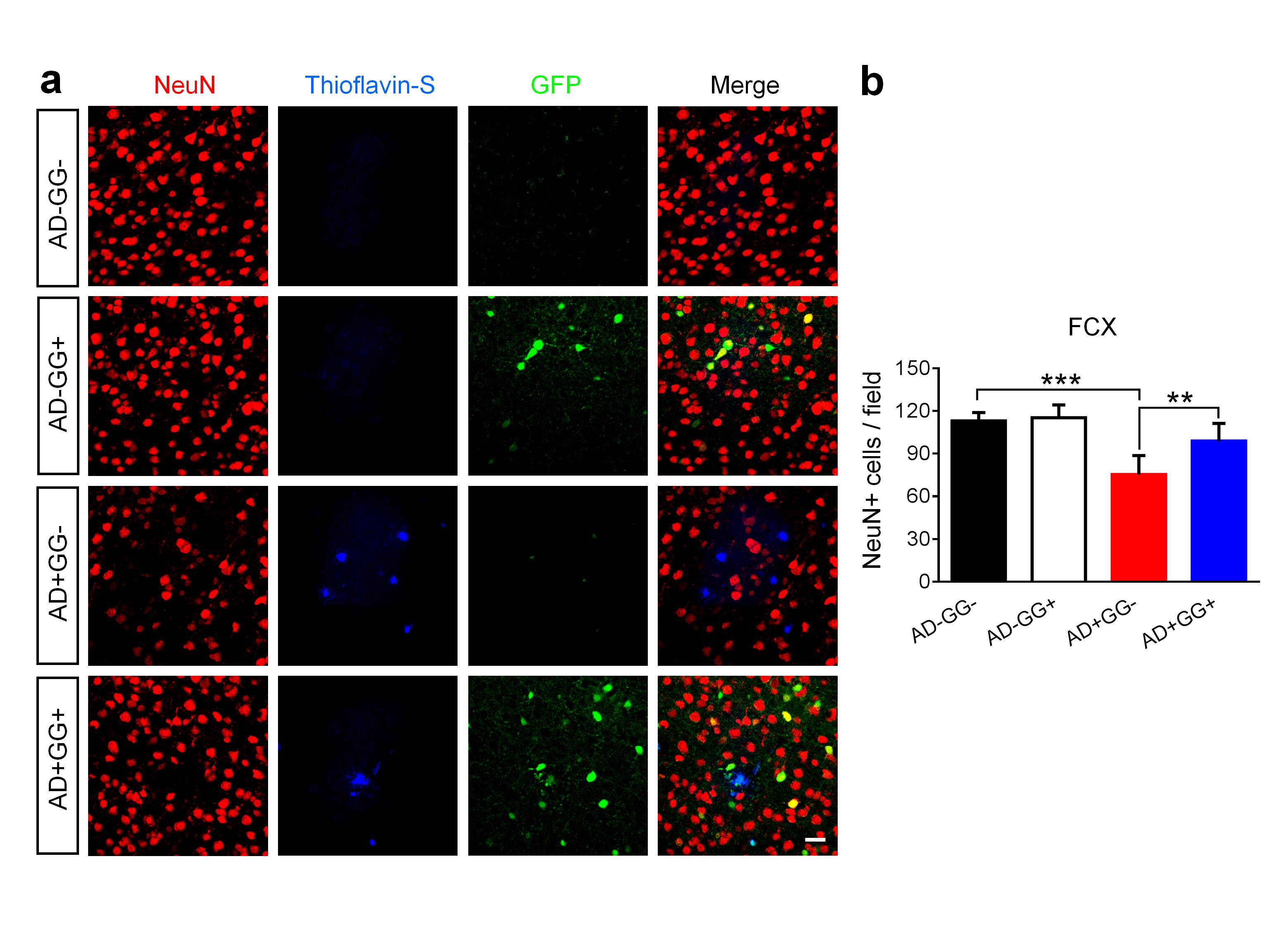


**Figure S2 Immunohistochemistry revealed less neuron loss in the frontal cortex region of 5xFAD mice with *Gad67* haploinsufficiency.** **a** Representative confocal images showing the distribution pattern of neuron (indicate by neuronal nuclear antigen NeuN, red) and amyloid-beta aggregates (indicated by thioflavin-s, blue). GFP positive cells were in green. Scale bar represents 30 μm. **b** Quantification data showing the NeuN positive cells number in each micrograph. N = 6 mice of 10-11 months age in each genotype group (AD-GG-, AD-GG+, AD+GG-, AD+GG+). Data are presented as mean ± s.e.m., * *p* < 0.05; ** *p* < 0.01; *** *p* < 0.001; one-way ANOVA with the Tukey’s post-hoc test when comparing multiple groups.


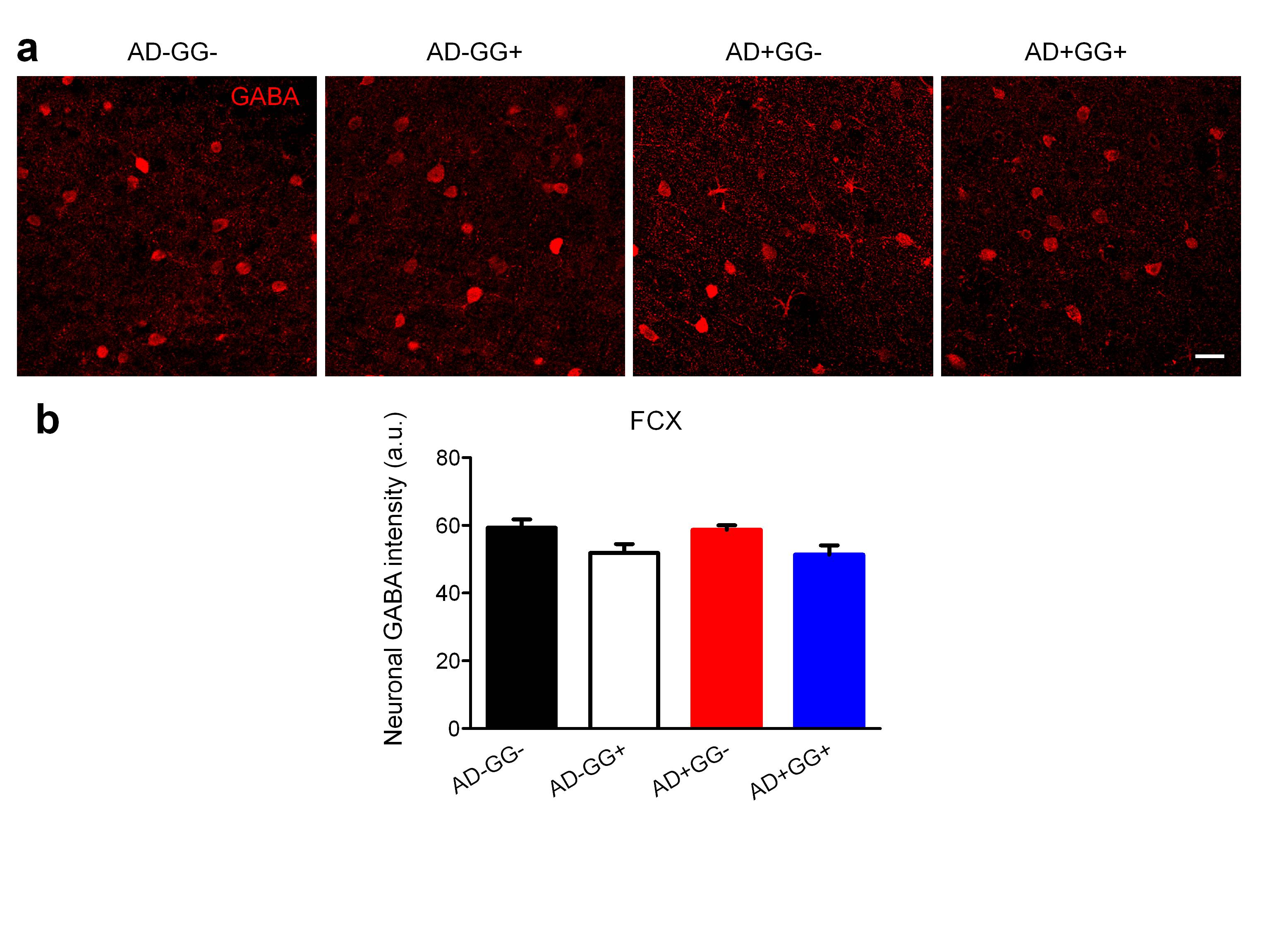


**Figure S3 Neuronal GABA content level remains similar in the frontal cortex among different genotypes of mice.** **a** GABA immunostaining (red) in frontal cortex region of AD-GG-, AD-GG+, AD+GG- and AD+GG+. Scale bar represents 30 μm. **b** Statistic graph reveals the similar level of neuronal GABA content in each genotype, no significant difference. N = 3 mice of 10-11 months age in each genotype group (AD-GG-, AD-GG+, AD+GG-, AD+GG+). Data are presented as mean ± s.e.m., one-way ANOVA with the Tukey’s post-hoc test when comparing multiple groups.


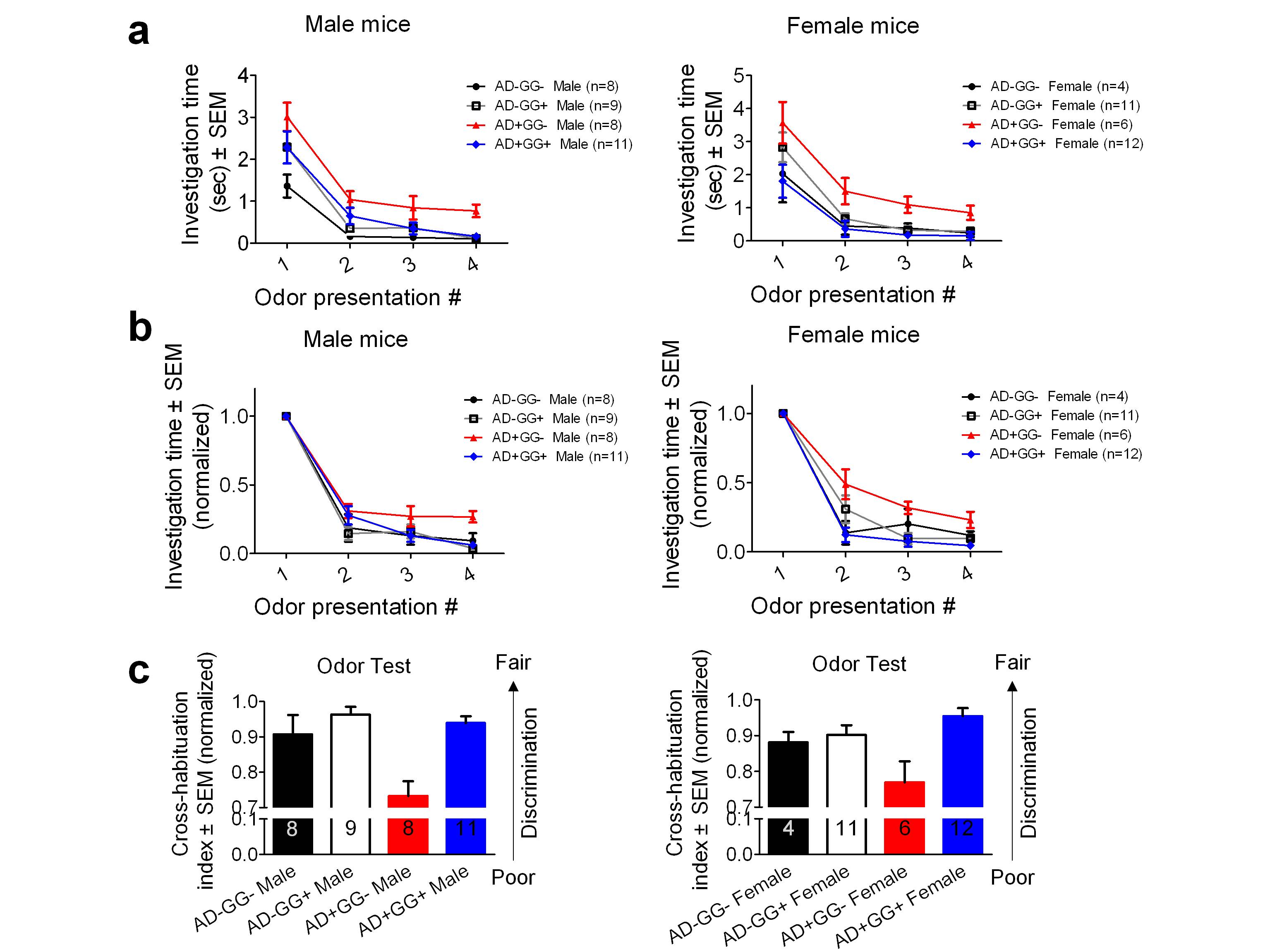


**Figure S4 Similar performance between male and female mice of the same genotype in the olfactory behavioral test.** **a** From left to right panel, the quantification graphs showing the investigation time of the male mice group and female mice group, respectively. **b** From left to right panel, the quantification graphs showing the investigation time (normalized) of the male mice group and female mice group, respectively. **c** From left to right panel, the statistic graphs reveal the cross-habituation index (normalized) for male mice group and female mice group, respectively. For the mice number of each group, refer to the labels in the each bar.

**Supplementary Table 1. Confocal imaging acquisition parameters**

| Olympus confocal microscope FV-1000 | Protein marker | Laser | Laser intensity | HV(high voltage) | Gain |
| --- | --- | --- | --- | --- | --- |
| Figure 1c | GAD67 | 543 | 68% | 773v | 1 |
| Figure 2, 3a and 3a | Aβ42 | 633 | 62% | 721v | 1 |
| Figure 4a | GABA | 543 | 68% | 703v | 1 |
| Figure 4a | GFAP | 633 | 62% | 591v | 1 |
| Figure 5a | Iba1 | 633 | 62% | 631v | 1 |
| Figure 5a | iNOS | 543 | 68% | 803v | 1 |
